# Cognition and Related Neural Findings on Methamphetamine Use Disorder: Insights and Treatment Implications From Schizophrenia Research

**DOI:** 10.3389/fpsyt.2019.00880

**Published:** 2019-12-17

**Authors:** Alexandre A. Guerin, Yvonne Bonomo, Andrew John Lawrence, Bernhard Theodor Baune, Eric J. Nestler, Susan L. Rossell, Jee Hyun Kim

**Affiliations:** ^1^Mental Health Theme, The Florey Institute of Neuroscience and Mental Health, Parkville, VIC, Australia; ^2^Florey Department of Neuroscience and Mental Health, University of Melbourne, Parkville, VIC, Australia; ^3^Department of Addiction Medicine, St Vincent’s Hospital, Melbourne, VIC, Australia; ^4^Department of Medicine, University of Melbourne, Melbourne, VIC, Australia; ^5^Women’s Alcohol and Drug Service, Royal Women’s Hospital, Melbourne, VIC, Australia; ^6^Department of Psychiatry, University of Melbourne, Melbourne, VIC, Australia; ^7^Department of Neuroscience, Friedman Brain Institute, Icahn School of Medicine at Mount Sinai, New York, NY, United States; ^8^Centre for Mental Health, Swinburne University of Technology, Melbourne, VIC, Australia; ^9^Department of Psychiatry, St Vincent’s Hospital, Melbourne, VIC, Australia

**Keywords:** methamphetamine use disorder, schizophrenia, cognition, memory, brain, MRI

## Abstract

Despite the prevalence of methamphetamine (meth) use disorder, research on meth is disproportionately scarce compared to research on other illicit drugs. Existing evidence highlights cognitive deficits as an impediment against daily function and treatment of chronic meth use. Similar deficits are also observed in schizophrenia, and this review therefore draws on schizophrenia research by examining similarities and differences between the two disorders on cognition and related neural findings. While meth use disorder and schizophrenia are two distinct disorders, they are highly co-morbid and share impairments in similar cognitive domains and altered brain structure/function. This narrative review specifically identifies overlapping features such as deficits in learning and memory, social cognition, working memory and inhibitory/impulse control. We report that while working memory deficits are a core feature of schizophrenia, such deficits are inconsistently observed following chronic meth use. Similar structural and functional abnormalities are also observed in cortical and limbic regions between the two disorders, except for cingulate activity where differences are observed. There is growing evidence that targeting cognitive symptoms may improve functional outcome in schizophrenia, with evidence of normalized abnormal brain activity in regions associated with cognition. Considering the overlap between meth use disorder and schizophrenia, targeting cognitive symptoms in people with meth use disorder may also improve treatment outcome and daily function.

## Introduction

Methamphetamine (meth) use disorder is defined by the Diagnostic and Statistical Manual of Mental Disorders 5th edition (DSM-5) as a substance use disorder characterized by compulsive meth-taking and -seeking despite serious negative consequences (1). Amphetamines are the second most used illicit drug in the world, second only to cannabis (2). Meth represents the majority of illicitly used amphetamines and is an urgent global health threat, with a rapidly increasing market (3). The Substance Abuse and Mental Health Services Administration estimated that close to 13 million people in the United States used meth in their lifetime (∼4% of the total population), with 569,000 people using meth in the past month (4). There is no government-approved medication to treat meth use disorder, and existing psychological interventions need much improvement in efficacy (5).

**Figure 1:**
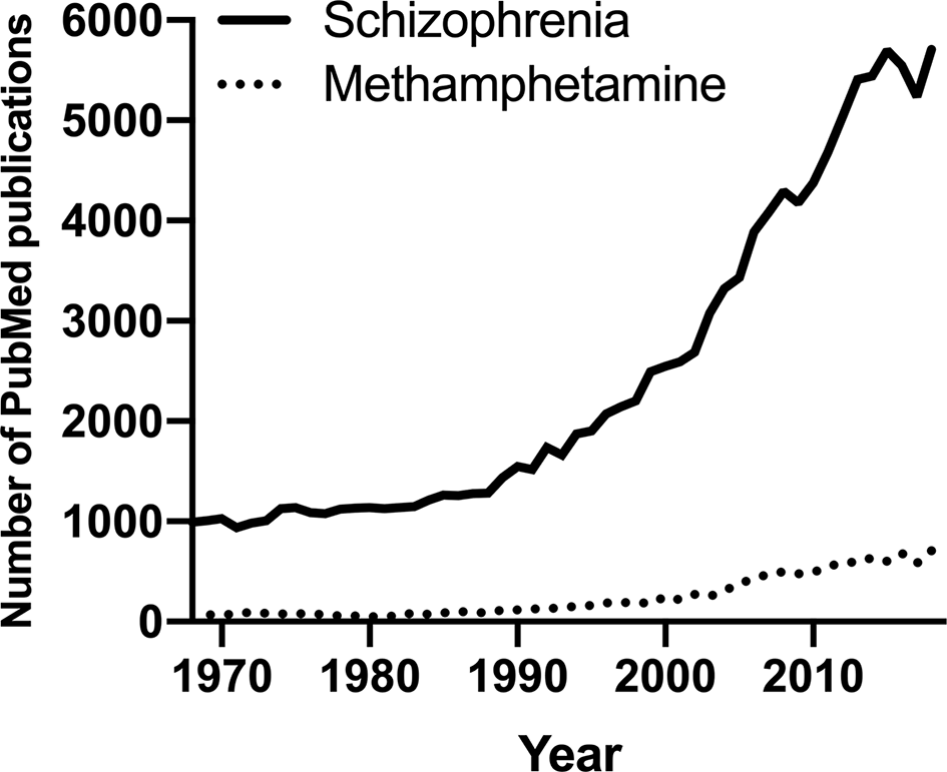
The number of PubMed publications in the last 50 years on schizophrenia or methamphetamine.

Despite the prevalence and associated harm, PubMed indicates that research on meth is disproportionately low compared to other substance use disorders (6) and other mental disorders. We thus propose to harness existing research in schizophrenia to provide much needed insight into meth use disorder to improve and innovate its therapeutics considering the evidence for their shared psychotic symptoms and genetic vulnerability (7–9). Schizophrenia is a chronic neuropsychiatric disorder characterized by disturbances in thought, perception, and behavior (1), and it is an extensively researched field with PubMed publications per year almost doubling all of illicit substance publications per year and more than ten times the number of publications on meth (**Figure 1**). Other than its wealth of existing data, schizophrenia is one of the best fields to leverage to understand meth use disorder because it is highly co-morbid with meth use (10). Meth is amongst the most used illicit substance in people with schizophrenia (11). Importantly, chronic meth use might promote the development of schizophrenia in at-risk individuals (12, 13), and a major animal model to capture some of schizophrenia-like psychotic symptoms relies on chronic meth exposure (14). For example, impairment in prepulse inhibition, a measurement of sensorimotor gating deficits often observed in people with schizophrenia (15), can be elicited following chronic meth exposure in rodents (16–19). To the best of our knowledge, no study has assessed sensorimotor gating impairment in people with meth use disorder, but rodent evidence suggests that chronic meth exposure in early life and adolescence may lead to long-lasting deficits in prepulse inhibition in adulthood in mice (20, 21).

Taken together, cognitive deficits and their associated neural dysfunction characterize both disorders (22–29). However, they have never been explicitly compared. The shared cognitive deficits are important to understand considering that many schizophrenia patients use psychostimulants as self-medication to reduce positive and negative symptoms, and improve mood states (30). Such efforts in turn may potentiate or exacerbate cognitive symptoms. Also, cognitive deficits are associated with poorer functional outcome in both disorders (31–33), hence existing effective therapies targeting cognitive deficits in schizophrenia may provide treatment avenues for similar deficits in meth use disorder.

The aim of this review is first to compare and contrast the cognitive impairments and related brain structure/activity between meth use disorder and schizophrenia. While many of the impairments are similar between the two, the associated neural changes can be different, which is important to understand the potential nuances in shared factors between the two disorders. We will then discuss approaches to treat cognitive symptoms in both disorders, with a focus on cognitive remediation therapy (CRT). The current review exclusively discusses studies in people with a DSM-IV or DSM-5 diagnosis of meth use disorder/dependence rather than to broadly include studies examining acute or casual meth users. This is to limit the substantial variability observed in findings due to large differences in meth intake between casual vs dependent users. Acute meth use and associated meth-induced psychosis-related cognitive deficits will not be discussed. Notably, there are limited studies that have directly compared people with meth use disorder and schizophrenia. We thus examined characteristics that were explicitly investigated in people with meth use disorder (**Table 1**) and then compared them against independent findings on schizophrenia. Where possible, studies that have directly compared the two disorders were highlighted.

**Table 1:** Summary of studies that have investigated cognitive deficits in meth use disorder, ordered in ascending order of shortest abstinence length reported in the sample. Percentage of female participant is specified if reported.

Study	Sample	n (% females)	Learning and Memory		Social Cognition		Working Memory		Inhibition & Impulsive Control	
			Types (measures)	Impaired in meth?	Types (measures)	Impaired in meth?	Types (measures)	Impaired in meth?	Types (measures)	Impaired in meth?
Dean et al. (34)	Healthy	17 (53%)					Verbal; Visual (LNS; SCAP)	**No**	Inhibition (Stroop color-word; CPT; ANT)	**Yes**
	Chronic meth users (current)	24 (50%)								
Lyoo et al. (35)	Healthy	120 (20%)							Inhibition (Stroop color-word)	**Yes**
	Chronic meth users (current)	106 (21%)								
Mahoney et al. (36)	Healthy	31 (45%)							Impulsivity (BIS)	**Yes**
	Chronic meth users (current)	31 (29%)								
Kim et al. (37)	Healthy	53 (23%)							Inhibition (Stroop color-word)	**Yes**
	Chronic meth users (current)	44 (20%)								
Andres et al. (38)	Healthy	34 (38%)							Impulsivity (BIS)	**Yes**, but only in current users
	Chronic meth users (current)	27 (44%)								
	Chronic meth users (abstinent 1 month–​24 years)	32 (37%)								
Su et al. (39)	Healthy	346 (∼60%)	Verbal (RBANS; OCL)	**Yes**						
	Chronic meth users (last use 1–7 days)	178 (∼18%)								
Simon et al. (40)	Healthy	65 (60%)	Verbal; Visual (repeated memory test)	**Yes** (verbal and visual)			Verbal (digit span)	**Yes**	Inhibition (Stroop color-word)	**Yes**
	Chronic meth users (last use within 3 days)	65 (45%)								
Simon et al. (41)	Healthy	40 (65%)	Verbal; Visual (repeated memory test)	**Yes** (verbal): **No** (visual)			Verbal (digit span)	**Yes**	Inhibition (Stroop color-word)	**Yes**
	Chronic meth users (last use within 3 days)	40 (50%)								
Monterosso et al. (42)	Healthy (smokers)	14 (28%)							Inhibition (SSRT)	**Yes**
	Healthy (non-smokers)	29 (67%)								
	Chronic meth users (last use 5–7 days)	11 (36%)								
Thompson (22)	Healthy	21 (52%)	Visual (repeated memory test)	**Yes**						
	Chronic meth users (last use 19 out of 30 days)	22 (32%)								
Iudicello et al. (43)	Healthy	Both times: 38 (8%)	Verbal; Visual (BVMT-R; HVLT-R)	Baseline & follow-up: **Yes** for non-abstinent users (visual, verbal)			Verbal (PASAT; LNS)	Baseline & follow-up: **No**		
	Chronic meth users (baseline: current; follow-up: non-abstinent)	Both times: 58 (9%)								
	Chronic meth users (baseline: current; follow-up: abstinent 1 year)	Both times: 25 (12%)								
Nestor et al. (23)	Healthy	18 (39%)							Inhibition (Stroop color-word)	**Yes**
	Chronic meth users (abstinent 4–7 days)	10 (50%)								
Simon et al. (44)	Healthy	Baseline: 28 (50%) Follow-up: 21 (43%)	Verbal; Visual (Repeated memory test; Selective reminder test)	Baseline & follow-up: **No**			Verbal; Visual (digit span; reading span; missing digit span)	Baseline & follow-up: **No**	Inhibition (Stroop color-word)	Baseline & follow-up: **No**
	Chronic meth users (baseline: abstinent 4–9 days; follow-up: abstinent 1 month)	Baseline: 27 (37%) Follow-up: 18 (28%)								
Kalechstein et al. (45)	Healthy	18 (17%)	Verbal; Visual (AVLT; WMS; CFT)	**Yes** (verbal); **No** (visual)			Verbal; Visual (LNS; VMS)	**No**	Inhibition (Stroop color-word)	**Yes**
	Chronic meth users (abstinent 5–14 days)	27 (30%)								
Schwartz et al. (24)	Healthy	44 (50%)							Impulsivity (DDT)	**Yes**
	Chronic meth users (abstinent 14–160 days)	61 (49%)								
Hoffman et al. (46)	Healthy	41 (27%)	Verbal (AVLT; Babcock story recall)	**Yes**					Inhibition (Stroop color-word)	**No**
	Chronic meth users (abstinent 0.5–6 months)	41 (24%)								
Woods et al. (47)	Healthy	71 (41%)	Verbal (HVLT-R)	**Yes**						
	Chronic meth users (abstinent 0.2–18.2 months)	87 (31%)								
Van Der Plas et al. (48)	Healthy	36 (47%)					Visual (Tic tac toe)	**Yes**		
	Chronic meth users (abstinent >15 days)	38 (66%)								
Boileau et al. (49)	Healthy	14 (21%)					Verbal (digit span)	**No**		
	Chronic meth users (abstinent ∼19 days)	16 (31%)								
Kim et al. (50)	Healthy	27 (0%)			Facial affect recognition (facial emotion recognition task; eye test; hitting task)	**Yes**				
	Chronic meth users (abstinent ∼20 days)	28 (0%)								
Uhlmann et al. (25)	Healthy	40 (28%)							Impulsivity (UPPS-P impulsive behavior scale)	**Yes**
	Chronic meth users (abstinent ∼21 days)	39 (28%)								
Uhlmann et al. (51)	Healthy	21 (19%)			Facial affect recognition; Theory of mind (emotion recognition task; mind in the eyes test)	**Yes**				
	Chronic meth users (abstinent ∼21 days)	21 (19%)								
Salo et al. (52)	Healthy	38 (45%)							Inhibition (Stroop color-word)	**Yes**, but only in early abstinence
	Chronic meth users (abstinent 3 weeks–​6 months)	41 (54%)								
	Chronic meth users (abstinent > 1 year)	28 (68%)								
Gonzalez et al. (53)	Healthy	19 (37%)					Verbal (digit span)	**Yes**		
	Chronic meth users (abstinent ∼30 day)	16 (25%)								
Morgan et al. (54)	Healthy	110 (36%)	Visual (BVMT-R)	**Yes**						
	Chronic meth users (abstinent 1.5–5 months)	114 (30%)								
Zhong et al. (32)	Healthy	Baseline: 58 (36%) Follow-up 1: 29 Follow-up 2: 25	Verbal; Visual (ISL; OCL)	Baseline & follow-up 1: **Yes** (verbal) **No** (visual) Follow-up 2: **No** (verbal & visual)	Facial affect recognition (social emotion cognitive task)	Baseline: **Yes** Follow-up 1 & 2: **No**	Visual (CPAL; 2-back task)	Baseline: **Yes** Follow-up 1 & 2: **No**		
	Chronic meth users (baseline: abstinent ∼1.5 months; follow-up 1: abstinent ∼4.5 months; follow-up 2: abstinent ∼7.5 months)	Baseline: 54 (26%) Follow-up 1: 44 Follow-up 2: 35								
Salo et al. (55)	Healthy	12 (0%)							Inhibition (Stroop color-word)	**Yes**
	Chronic meth users (abstinent 2–4 months)	8 (0%)								
Salo et al. (56)	Healthy	16 (50%)							Inhibition (Stroop color-word)	**Yes**
	Chronic meth users (abstinent 2–12 months)	12 (58%)								
Kim et al. (57)	Healthy	20 (25%)							Inhibition (WCST)	**Yes**
	Chronic meth users (abstinent 2.6–30.6 months)	29 (7%)								
Rendell et al. (58)	Healthy	20 (40%)	Verbal; Visual (AVLT; virtual week)	**Yes**			Verbal (digit span)	**Yes**		
	Chronic meth users (abstinent 3–8 months)	20 (40%)								
Henry et al. (59)	Healthy	20 (40%)	Verbal (AVLT)	**Yes**	Facial affect recognition; Theory of mind (facial affect test; mind in the eyes test)	**Yes**				
	Chronic meth users (abstinent 3–8 months)	20 (40%)								
Johanson et al. (60)	Healthy	18 (33%)	Verbal (CVLT)	**Yes**			Visual (SWM; DMS)	**No**		
	Chronic meth users (abstinent 0.25–18 years)	16 (31%)								
Iudicello et al. (61)	Healthy	26 (8%)	Prospective (MIST)	**Yes**						
	Chronic meth users (abstinent ∼105 days)	39 (58%)								
Cherner et al. (62)	Healthy	46 (50%)	Verbal; Visual (BVMT-R; HVLT-R; story memory test; figure memory test)	**Yes**			Verbal (PASAT; LNS)	**No**	Inhibition (Stroop color-word)	**No**
	Chronic meth users (abstinent ∼4 months)	54 (26%)								
Rippeth et al. (63)	Healthy	60 (50%)	Verbal; Visual (BVMT-R; HVLT-R)	**Yes**			Verbal (PASAT; LNS)	**Yes**		
	Chronic meth users (abstinent ∼4.5 months)	47 (36%)								
Chang et al. (64)	Healthy	20 (50%)	Verbal (AVLT)	**No**			Visual (One-back task)	**No**		
	Chronic meth users (abstinent 6–8 months)	20 (50%)								
King et al. (65)	Healthy	74 (50%)							Inhibition (Stroop color-word)	**Yes**
	Chronic meth users (abstinent ∼252 days)	54 (68%)								
Stock et al. (66)	Healthy	32					Verbal; Visual (digit span; Corsi block span)	**No**	Inhibition (Stroop color-word)	**Yes**, but only in early abstinence
	Chronic meth users (abstinent ∼9.9 months)	13 (38%)								
	Chronic meth users (abstinent ∼47.6 months)	14 (43%)								
Salo et al. (26)	Healthy	30 (43%)							Inhibition (Stroop color-word)	**Yes**
	Chronic meth users (abstinent ∼13.7 months)	30 (50%)								
Gonzalez et al. (67)	Healthy	41 (51%)	Verbal; Visual (HVLT-R; BVMT-R; story memory test; figure memory test)	**Yes**			Verbal (PASAT, LNS)	**No**		
	Chronic meth users (abstinent up to 18 months)	26 (46%)								
Salo et al. (68)	Healthy	16 (50%)							Inhibition (Stroop color-word)	**Yes**
	Chronic meth users (abstinent ∼20 months)	36 (64%)								
Salo et al. (69)	Healthy	17 (47%)							Inhibition (Stroop color-word)	**Yes**
	Chronic meth users (abstinent ∼20.98 months)	37 (65%)								
Moon et al. (70)	Healthy	18 (0%)	Verbal; Visual (AVLT; CFT)	**Yes** (verbal); **No** (visual)						
	Chronic meth users (abstinent ∼1.79 years)	19 (0%)								

ANT, attention networks task; AVLT, auditory verbal learning test; BIS, Barratt impulsiveness scale; BVMT-R, brief visuospatial memory test—revised; CFT, complex figure test; CPAL, continuous paired association learning task; CPT, continuous performance test; CVLT, California verbal learning test; DDT, delayed discounting task; DNM, delayed non-match to sample task; DMS, delayed match to sample; HVLT-R, Hopkins verbal learning test—revisited; ISL, international shopping list task; LNS, letter-number sequence; MIST, memory for intentions screening test; OCL, one card learning task; PASAT, paced auditory serial addition task; RBANS; repeatable battery for the assessment of neuropsychological status; SCAP, spatial capacity delayed response test; SSRT; stop-signal reaction time; SWM, spatial working memory span; VMS, visual memory span; WCST, Wisconsin card sorting test; WMS, Wechsler memory scale.

## Cognitive Deficits and Related Brain Dysfunction

Meth specifically acts on dopamine release by disrupting intravesicular pH and reversing transport of dopamine *via* plasma membrane transporters, which impairs the uptake of dopamine and its concentration within synaptic vesicles. The result is higher cytosolic concentrations of dopamine in nerve terminals, which leads to excess dopamine concentration in the synaptic cleft (71–73). This likely leads to lasting neuroadaptations (74) to affect cognition. Subcortical hyperdopaminergia and prefrontal hypodopaminergia is hypothesized to be part of the pathophysiology of schizophrenia (75), which may cause neural dysfunction associated with cognition overlapping with chronic meth use. A meta-analysis reported that people with meth use disorder show deficits of medium effect size in cognition (76). In people with schizophrenia, impairments of medium to large effect sizes are observed in similar domains (77).

The scope and breadth of cognition studies in meth use disorder are severely lacking compared to schizophrenia. Thus, the present review specifically highlights dominantly studied aspects of cognition in chronic meth use, namely learning and memory, social cognition, and two key executive functions: working memory and inhibitory/impulsive control. The findings are then compared against corresponding studies in schizophrenia.

### Learning and Memory

While all types of memory are not yet assessed in meth use disorder, there is strong evidence that both current and abstinent meth users display mild impairments in visual, verbal, and prospective learning and memory (22, 32, 39–41, 43–47, 54, 58–63, 67, 70), even observed after 1.8 years of abstinence (**Table 1**). People with schizophrenia display severe impairments in similar domains examined using the same tests (78–80). Prospective memory impairments are of particular interest because they are negatively associated with treatment outcomes due to poorer adherence to medication regimens (81) and greater likelihood of missed appointments (79).

While it is difficult to establish meth dependence in animals, preclinical rodent studies using chronic meth exposure (minimum 7 days of exposure) suggest a long-lasting causative effect of meth on different types of learning and memory. Meth self-administrating rats display both short- and long-term impairments in recognition memory (82, 83). In contrast, experimenter-led chronic injections of meth only impairs long-term recognition memory (84). In a study investigating the effect of meth on auditory associative learning and memory, experimenter-led chronic injections of meth disrupted recall of inhibitory memory, whereas meth self-administration disrupted associative learning (85). Spatial memory impairments are also observed in self-administrating rats and rats subject to experimenter-led chronic injections (86–88), with effects lasting up to 3 weeks following abstinence (89). In contrast, a study by Kesby and colleagues (90) found that experimenter-led chronic meth injections may improve learning in mice in a visual discrimination task (90). Taken together, it may be that chronic meth injections may initially improve learning processes but lead to deficits in the long-term, whereas meth self-administration consistently lead to memory impairments.

Learning and memory processes rely on the prefrontal cortex (PFC) and medial temporal lobe including the hippocampus (91). Altered structure and activity of these regions have been described in both meth use disorder and schizophrenia that may be associated with poorer memory. For example, a MRI study showed an association between decreased bilateral hippocampal volume and poorer performance on a word-recall task in current meth users (22), although a recent study with bigger sample size failed to find a link between visual/verbal memory and hippocampal volume in abstinent meth dependents (92). In schizophrenia, the association between decreased bilateral hippocampal volume and poorer performance on a verbal recall task is well established (93). This suggests a shared role for hippocampal volume reduction in verbal learning and memory impairments in both disorders.

No study has investigated the neural correlates of prospective memory performance in people with meth use disorder, but there is one study in people with schizophrenia. Chen et al. (29) found that compared to healthy controls, people with schizophrenia displayed hypoactivity in the frontal, parietal and temporal cortex (29). Indeed, prospective memory performance and activation of the rostral PFC and parietal lobe are positively associated in healthy people (94). People with meth use disorder or schizophrenia display reduced gray matter in the parietal lobe (35, 95, 96,), which may explain the visual learning and memory deficits observed. In addition, parietal gray matter reduction is observed in people with childhood-onset schizophrenia (95) and adolescents with meth use disorder (35). These findings suggest a role for parietal lobe that may be an early-onset risk factor for both disorders that may be targeted for treatment in childhood/adolescence.

### Social Cognition

A recent meta-analysis in people with meth use disorder found that social cognition impairments were amongst the largest cognitive deficits observed, specifically in theory of mind (ToM) and emotion processing (76), which are also widely described in schizophrenia (97, 98). There is mice evidence suggesting a link between chronic meth injections during mid-late adolescence and disruption of social interaction following 2 weeks of abstinence in males (99). While social cognition deficits are apparent in current and short-term abstinent meth dependents (32, 50, 51), there is conflicting evidence following long-term abstinence. For example, Henry et al. (59) found impairments of large effect size in participants with meth use disorder who have been abstinent for 3-8 months compared to healthy controls (59), whereas Zhong et al. (32) observed no differences after 7.5 months of abstinence (32). Given that social cognition deficits have a significant negative impact on social and vocational functioning in people with schizophrenia (100, 101), it is clear that further research of the deficit and its functional implications is warranted in people with meth use disorder.

Key brain structures underlying social cognition include ventro- and dorsolateral PFC (vlPFC and dlPFC), orbitofrontal cortex, anterior cingulate cortex (ACC), insular cortex, and amygdala (102–104). Indeed, alterations in those regions underlying social cognition are observed in meth use disorder and schizophrenia. Compared to healthy controls, there is reduced activation of the vlPFC and dlPFC in meth dependents (105, 106) and people with schizophrenia (107, 108) in response to threatening or fearful faces. Such prefrontal dysfunction may indicate failure to integrate socio-emotional information (109). People with schizophrenia also display hypoactivity of the cingulate cortex in response to negative words (107). In contrast, *hyperactivity* of the ACC and posterior cingulate cortex (PCC) is associated with response to negative emotions in people with meth use disorder (105, 106). Considering that ACC hyperactivity is linked to hypersensitivity to threat (106) and PCC hyperactivity is linked with recollection of past negative memories (105), it may be that emotional processing deficits arises from hyposensitivity to threat/sadness in schizophrenia but hypervigilance/distraction to threat/sadness in meth use disorder. This has important treatment implications and should be investigated.

Bilateral insular hypoactivity is observed in people with meth use disorder when presented with fearful and threatening images (105). Similarly in schizophrenia, left insular hypoactivity is associated with happy and fearful facial expression processing (110, 111), and disgust facial expressions in non-paranoid people with schizophrenia (112). Left insular hypoactivity when presented with sad faces is associated with adolescent-onset schizophrenia (113), which suggests that insular hypoactivity to sad faces may also be involved in adolescent-onset meth use. Consistent with functional MRI (fMRI) findings, insular cortex gray matter volume reduction is also observed in people with meth use disorder (24, 114, 115) or schizophrenia (95, 116). In fact, a meta-analysis revealed that insular gray matter showed the largest decrease of all brain regions in people with schizophrenia (96), with larger volume reduction in the anterior compared to the posterior insular cortex (117). Such insular abnormalities in both disorders may be linked to decreased empathy and abnormal response to threatening situations (105).

While a recent neuroimaging meta-analysis revealed large decreases in insular and bilateral medial PFC activation during ToM tasks in people with schizophrenia (118), no studies have investigated the neural correlate of ToM impairments in people with meth use disorder. ToM deficits appear consistent in meth use disorder, thus it would be interesting to examine whether its neural correlates are shared with people with schizophrenia.

### Executive Functions: Working Memory and Inhibitory Control

Executive functions are high-order cognitive processes necessary to balance new ideas, think before acting, remain focused, and resist temptations to ultimately control behaviors such as decision making (119). Such cognitive processes include inhibitory control, working memory, attentional control, and cognitive flexibility. Moderate to severe impairments in working memory and inhibitory control have been described in meth use disorder and schizophrenia (76, 120–123). Longitudinal evidence suggests that executive function impairments may predispose individuals to developing schizophrenia (124). While there is no such study in people with meth use disorder, a rodent study showed that reduced executive function leads to increased meth self-administration (125), suggesting that individual differences in executive function may contribute to the development and maintenance of meth dependence. This review will focus working memory and inhibitory control because other types of executive function have not been as thoroughly assessed in people diagnosed with meth use disorder.

#### Working Memory

Some studies have reported an association between working memory impairments and meth dependence in both current (40, 41, 53) and abstinent (32, 43, 48, 58, 62, 63, 66) chronic meth users, whereas others have found no association (44, 45, 49, 60, 64, 67). Although this may be due to different periods of abstinence across studies, inconsistent findings are still observed across studies with similar length of abstinence (**Table 1**). Additionally, some of the strongest effects of meth use on working memory were found in polydrug users (53, 63). Nevertheless, a meta-analysis recently revealed a moderate overall deficit in working memory in meth use disorder (76). In addition, male rats receiving chronic meth injections show long-lasting impairments in spatial working memory (126, 127). Given that impaired working memory in meth users is associated with disrupted social adaptation, global functioning, and unemployment (31, 32), more research is needed to elucidate whether such deficits are a predictor for the development of meth use disorder or a consequence of chronic meth use. In schizophrenia, working memory deficits form a core feature (120, 121), and premorbid working memory may be one of the most prominent factors predisposing individuals to developing the disorder (124, 128).

Studies focusing on working memory processes observed both hyper- and hypoactivation of the dlPFC in people with schizophrenia (27). Discrepancies between studies may to be due to varying task difficulty across studies (27), suggesting an association between dlPFC activation and degree of working memory impairments. We are not aware of an fMRI investigation during working memory tasks in people with meth use disorder. However, a perfusion MRI study found a positive correlation between working memory performance and regional cerebral flow in the left temporoparietal region and in the right lateral parietal cortex of abstinent meth users (64). Given that not all people with meth use disorder display impairments in working memory (**Table 1**), it would be informative to investigate individual patterns of frontal lobe dysfunction associated with such deficits and investigate whether a hyper- or hypoactivity emerges.

#### Inhibitory Control

The Stroop task, which measures the ability to suppress irrelevant information, is one of the most commonly used tests of inhibitory control in neuropsychiatric patients. Chronic meth use is associated with poorer performance in the Stroop task in adolescents (35, 37, 65) and adults with meth use disorder (23, 26, 34, 40, 41, 45, 66–52). Effects in adolescents suggest that reduced inhibitory control may be a predisposing factor to developing meth use disorder. There is extensive evidence that schizophrenia also leads to poorer performance on the Stroop task compared to controls (122, 123). Salo et al. (2011) explicitly compared Stroop performance between people with schizophrenia and people with meth use disorder, and found greater inhibitory deficits in early abstinent (2–7 days) meth dependents compared to schizophrenia patients (52). This suggests that withdrawal from meth may contribute to the severity of cognitive symptoms because the inhibition deficits and withdrawal symptoms decreased over time (52). Inhibitory control and impulsivity are associated (129). Indeed, people with schizophrenia or meth use disorder both display poor impulse control (24, 25, 36, 38, 42, 130, 131). Notably, impulse-related functions are among the most impaired in meth use disorder (76), and poor impulsivity is regarded as one key predisposing factor to developing substance use disorder (132). In addition, Monterroso et al. (42) found that reaction time in an impulse control task positively correlates with grams of meth used per week, highlighting the relationship between poor impulse control performance and the extent of recent meth use (42). These deficits have important clinical implications. In meth use disorder, impaired inhibition is linked with unemployment (31), and poor impulse control is associated with treatment non-completion (133) and relapse (134). In schizophrenia, poorer inhibition and greater impulsivity have a negative impact on daily function (135, 136). In rodents, chronic meth injections lead to an age-dependent impairment in inhibition (78, 130). Interestingly, this effect is not observed after a week of withdrawal (131), suggesting that abstinence may reverse deficits in inhibition.

Inhibitory control impairments correlate with reduced gray matter volume in the middle frontal gyrus in meth use disorder (57). In schizophrenia, a reduction in orbital inferior frontal gray matter is observed (137). Disrupted frontal white matter integrity is linked with impulsivity (25) and inhibition (35) in meth use disorder. Meth dependents also display structural abnormalities in the genu of the corpus callosum (138, 139), a white matter tract which carries fibers originating from the PFC. Poorer corpus callosum integrity is associated with impaired inhibition (140, 141) and impulse control (38). In addition to structural abnormalities, people with meth use disorder display reduced activation of the PFC when performing the Stroop task (56), more specifically in the right inferior frontal gyrus (IFG), ACC (23) and the PFC (26). Likewise, people with schizophrenia display hypoactivity of the right IFG, ACC, and PFC when performing similar tasks (28, 142). Hypoactivity in the ACC and right IFG is also observed in people with early onset schizophrenia (143). It would be important to also examine these brain regions during executive function tasks in people with adolescent- vs adult-onset of meth use disorder.

Metabolic alteration of the ACC is also associated with impaired inhibitory control in both disorders, which is measured by levels of *N*-acetylaspartate, a marker of neuronal integrity (144). Reduced *N*-acetylaspartate levels in the ACC are observed in meth use disorder (145) and schizophrenia (146–149). *N*-acetylaspartate levels correlate with poorer attention and inhibition in adult (68) and adolescent (37) meth users, and people with schizophrenia (150–152). Interestingly, reduced *N*-acetylaspartate in ACC is more dramatic with early onset of meth use (37), and reduced ACC levels of *N*-acetylaspartate are present at the early stages of schizophrenia (149), and in high-risk offspring of schizophrenia patients (152).

**Figure 2:**
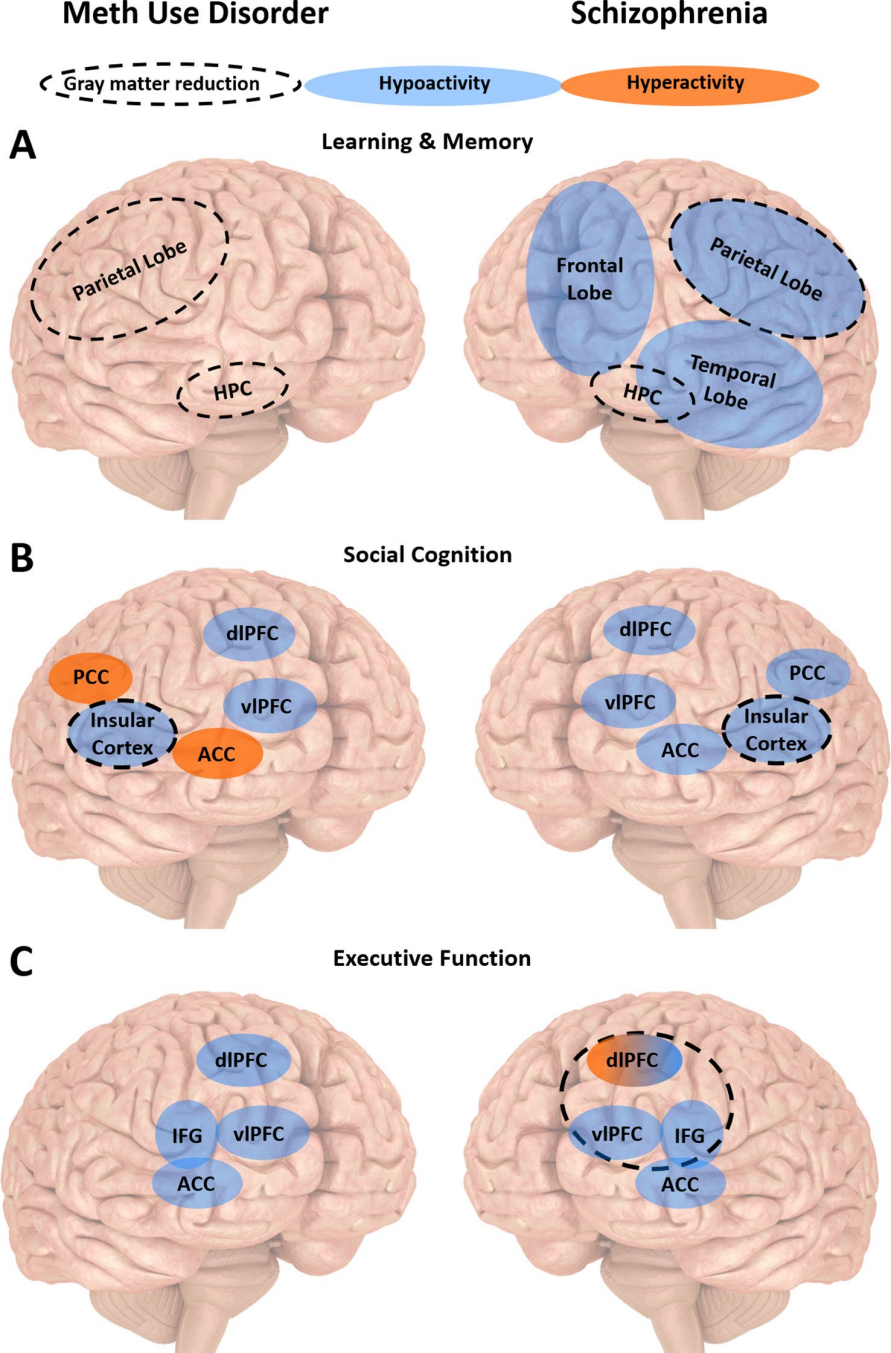
Summary of the neurobiology underlying cognitive impairments in meth use disorder and schizophrenia. **(A)** People with meth use disorder or schizophrenia display similar patterns of gray matter reduction associated with learning and memory impairments in the hippocampus (HPC) and parietal lobe. People with schizophrenia also display reduced activity in the frontal, parietal and temporal lobes. **(B)** People with meth use disorder or schizophrenia display similar patterns of gray matter reduction associated with social cognition impairments in the insular cortex. They also display reduced activity in the dorsolateral and ventrolateral prefrontal cortices (dlPFC and vlPFC, respectively). They display opposite patterns of activity in the anterior and posterior cingulate cortices (ACC and PCC, respectively). **(C)** People with meth use disorder or schizophrenia display similar reduced activity in the vlPFC, ACC and inferior frontal gyrus (IFG) associated with executive dysfunction. People with schizophrenia may display dlPFC hypo- or hyperactivity, depending on task difficulty and working memory load. Executive dysfunction is also associated with reduced medial frontal gyrus (MFG) gray matter in people with meth use disorder, and reduced orbital IFG gray matter in people with schizophrenia.

Taken together, impairments in learning and memory, social cognition, working memory and inhibitory control are observed in schizophrenia and meth use disorder. Such deficits affect treatment completion and outcomes in both conditions (31, 32, 79, 81, 100, 135, 136). While there is evidence that cognitive deficits may be a risk factor for the development of schizophrenia, it remains unclear whether they predispose an individual towards meth dependence or are the result of chronic meth use. Brain studies suggest that people with schizophrenia and meth use disorder display largely similar patterns of structural and functional brain abnormalities in regions involved in key cognitive processes, with the exception of brain regions underlying emotion regulation (**Figure 2**). Such abnormalities may predict disease progression. What is clear from all the existing studies of cognition and related brain regions in meth use disorder is how much the findings overlap with corresponding studies in schizophrenia, with far more similarities compared to differences between the two disorders. This is in contrast to the many symptomatic differences between the two disorders (7, 153). While we do not know the mechanisms underlying such similarities in cognitive deficits and brain dysfunction, such overlaps provide an important opportunity to consider employing existing schizophrenia therapies for people with meth use disorder.

## Interventions to Improve Cognition in Meth Use Disorder: Insights From Schizophrenia Research

The use of various pharmacotherapies to improve cognition in people with schizophrenia have yielded mixed results. A meta-analysis revealed that medication targeting the cholinergic system result in marginal improvement in verbal and spatial learning and memory (154). Preliminary evidence also suggest that oxytocin may improve social cognition and verbal learning and memory (155), but the efficacy of such intervention has proven to be inconclusive and more research is still needed (101). Results from a large scale multisite study suggests that treatment with antipsychotic medication only results in limited cognitive improvement (156). In addition to pharmacological treatments, a recent systematic review found that repeated transcranial magnetic stimulation may be beneficial in improving verbal and working memory, but not other functions such as cognitive flexibility (157).

On the other hand, CRT has shown promising results in the alleviation of cognitive deficits, with several meta-analyses revealing improvement of medium effect sizes in people with schizophrenia (158, 159). The Cognitive Remediation Experts Workshop (Florence, Italy, 2010) defines CRT as a “behavioral training-based intervention that aims to improve cognitive processes with the goal of durability and generalization”. A range of CRTs have been developed over the past 50 years to target cognitive symptoms specifically in schizophrenia, with CRT well received by participants (160, 161). CRTs use diverse methods, such as drill and practice exercises, cognitive strategies training, and group discussions (159, 162). Consistent with the present review’s observations that cognitive deficits overlap in schizophrenia and meth use disorder, preliminary evidence suggests that CRTs are also beneficial in people with meth use disorder (163, 164). Especially exciting are the results of two meta-analyses in schizophrenia patients that revealed CRT increased activation of brain regions affected in meth use disorder such as the dlPFC, mPFC, parietal cortex, insula, and thalamus (165, 166), and increased white matter integrity of the corpus callosum was also observed (167), highlighting that CRT may address the neurobiology underlying cognitive impairments. Overall, CRT has been shown to more consistently improve cognition in people with schizophrenia compared to other therapeutics avenues, with effect sizes varying between domains. CRT will therefore be the focus of this review.

### Targeting Learning and Memory Deficits

Improvements in verbal memory are observed in people with schizophrenia following CRT (158, 159). There is also evidence that cognitive training may improve event-based prospective memory in people with schizophrenia (80). Although prospective memory training appears to be a promising treatment approach, effect on daily function and functional outcome has yet to be investigated in schizophrenia.

To the best of our knowledge, similar memory training in people with meth use disorder has not yet been described. Given the negative impact of poor prospective memory on treatment outcome, it would be beneficial to consider developing CRT targeting such deficits in meth use disorders. CRT focusing on verbal and prospective memory training may be the most effective to treat meth use disorder because visual learning and memory is one of the few cognitive domains failing to respond to CRT (158, 159). Considering the overlapping link between verbal memory and hippocampal volume, frontal and parietal lobe function in both disorders, it would be informative to investigate whether CRT affects structure and function of those regions.

### Targeting Social Cognition Deficits

Bechi and colleagues (168) showed that combination of CRT and social cognitive or ToM training improved social cognitive abilities even further than CRT alone in people with schizophrenia (168). ToM training involves reading comic strips to be trained to recognize relevant details and collect and meaningful pieces of information such as place, time, characters’ actions, and physical features. Another study revealed that adding to standard CRT computerized social cognition training such as the interactive guide to emotions that is designed to train patients to recognize emotions and other mental states, produced greater improvement not only in social cognition, but also other cognitive domains such as visual memory and executive function in people with schizophrenia (169). Evidence reviewed by Campos et al. (170) revealed that emotion recognition training leads to an increase in activation in the fronto-temporal-occipital regions, postcentral gyrus, right amygdala, medial PFC, and right putamen in people with schizophrenia (170). Hyperactivity in those regions correlated with social cognition improvement, in particular medial PFC activation was associated with increased social functioning 6 months after treatment (170). Such evidence in schizophrenia suggests emotion recognition training as a strong candidate to improve social cognition in people with meth use disorder. However, interventions specifically targeting social cognition have not yet been studied in people with meth use disorder. Note that there is an opposite pattern of cingulate dysfunction underlying emotion regulation observed in people with schizophrenia and meth use disorder (**Figure 2**). It is possible that there is a dissociation between the two disorders in how emotion recognition training affects the cognitive deficits and the related hyperactivity in the cingulate cortex.

### Targeting Working Memory Deficits

Evidence reviewed by Lett et al. (171) suggests that computer-based programs using auditory exercises aiming to improve the speed and accuracy of auditory information processing produce long-lasting improvement in verbal working memory in people with schizophrenia (171). This is in line with a recent meta-analysis specifically investigating computer-based drill and practice training (172). Prikken et al. (172) found that working memory was among the most improved domains, and noted that shorter, but more intense training programs yielded larger effect sizes (172). On the other hand, they found limited improvements in functional outcome (172), which suggests that computerized training programs should be used in conjunction with another line of CRT involving face-to-face training.

Consistent with schizophrenia research, a pilot study in people with meth use disorder found improvement in working memory following four weeks of increasingly difficult N-back memory task training, which was also linked to improved impulse control (164). In people with meth use disorder, it is promising that working memory training has been shown to normalize frontostriatal structure and function (163).

## Summary

Meth use disorder and schizophrenia are two distinct but often comorbid mental disorders. The present review highlights shared cognitive impairments and brain abnormalities in people with schizophrenia or meth use disorder, with the hope to gain insight from schizophrenia research to develop treatments for people with meth use disorder, which is a global problem with increasing health, social and economic burden (173). Their shared key features including deficits in learning, memory, social cognition, working memory and inhibitory control, and abnormal frontostriatal and insular cortex structure and function, all impact on treatment outcome and daily functioning. There is some evidence that these deficits and abnormalities may precede the development of the disorders. Targeted treatment of the cognitive deficits in a vulnerable population may improve brain and cognition, and prevent or delay the onset of the disorders. Such treatment approaches for meth use disorder can capitalize on the well-established literature on schizophrenia. Specifically CRTs have been shown to successfully improve cognitive impairments, normalize brain function, and increase treatment efficacy in people with schizophrenia, and these treatment approaches should be examined for their efficacy to improve similar impairments in people with meth use disorder. This is an urgent call to action because there is no FDA-approved pharmacotherapy to treat stimulant use disorders. Importantly, more research is needed to fully understand the mechanisms underlying CRT, with the aim to tailor CRT for each individual patient with different levels of cognitive and brain impairments that have been shown to affect treatment outcomes.

## Author Contributions

AG, SR, and JK contributed to the conception and design of the review. AG and JK conducted the review. AG wrote the initial version of the manuscript, with subsequent contribution from YB, AL, BB, EN, SR, and JK. All authors contributed to and approved the submitted version.

## Funding

This research was supported by a Melbourne Research Scholarship from the University of Melbourne (AG); National Health and Medical Research Council (NHMRC) Principal Research Fellowship (AL); National Institutes of Health Grant P01DA008227 (EN); and NHMRC Career Development Fellowship (JK).

## Conflict of Interest

The authors declare that the research was conducted in the absence of any commercial or financial relationships that could be construed as a potential conflict of interest.

The reviewer EL declared a shared affiliation, with no collaboration, with one of the authors EN to the handling editor.
